# Is there a divide between local medicinal knowledge and Western medicine? a case study among native Amazonians in Bolivia

**DOI:** 10.1186/1746-4269-4-18

**Published:** 2008-08-18

**Authors:** Laura Calvet-Mir, Victoria Reyes-García, Susan Tanner

**Affiliations:** 1 Institut de Ciència i Tecnologia Ambientals, Universitat Autònoma de Barcelona, 08193 Bellatera, Barcelona, Spain; 2 ICREA and Institut de Ciència i Tecnologia Ambientals, Universitat Autònoma de Barcelona, 08193 Bellatera, Barcelona, Spain; 3 Heller School for Social Policy and Management, Brandeis University, Waltham, MA, 02454-9110, USA; 4 Department of Anthropology, University of Georgia, Athens, GA, 30602, USA

## Abstract

**Background:**

Interest in ethnomedicine has grown in the last decades, with much research focusing on how local medicinal knowledge can contribute to Western medicine. Researchers have emphasized the divide between practices used by local medical practitioners and Western doctors. However, researchers have also suggested that merging concepts and practices from local medicinal knowledge and Western science have the potential to improve public health and support medical independence of local people. In this article we study the relations between local and Western medicinal knowledge within a native Amazonian population, the Tsimane'.

**Methods:**

We used the following methods: 1) participant observation and semi-structured interviews to gather background information, 2) free-listing and pile-sorting to assess whether Tsimane' integrate local medicinal knowledge and Western medicine at the conceptual level, 3) surveys to assess to what extent Tsimane' combine local medicinal knowledge with Western medicine in actual treatments, and 4) a participatory workshop to assess the willingness of Tsimane' and Western medical specialists to cooperate with each other.

**Results:**

We found that when asked about medical treatments, Tsimane' do not include Western treatments in their lists, however on their daily practices, Tsimane' do use Western treatments in combination with ethnomedical treatments. We also found that Tsimane' healers and Western doctors express willingness to cooperate with each other and to promote synergy between local and Western medical systems.

**Conclusion:**

Our findings contrast with previous research emphasizing the divide between local medical practitioners and Western doctors and suggests that cooperation between both health systems might be possible.

## Background

In recent years researchers and policy-makers have shown a growing interest in the knowledge held by indigenous peoples. The interest has been partially driven by research that suggests that indigenous knowledge might be the key to inform conservation efforts and local empowerment and development [1-4].

Researchers and policy-makers have debated the relations between indigenous knowledge and Western science under the assumption that merging multiple epistemologies might improve the well-being of indigenous people [5,11,15-17]. Some researchers consider knowledge held by indigenous people as a form of knowledge that contrasts with the knowledge produced by Western science [5-10], but others argue that there is a significant overlap, and that the two do not necessarily oppose each other [11-14]. What we lack are studies focussing on how indigenous peoples conceptualize the relations between indigenous knowledge and knowledge produced by Western science, and how they use those to forms of knowledge in their daily life.

Understanding how indigenous peoples conceptualize local and Western science matters for the wellbeing of indigenous peoples [18-22]. Research suggests that local medicinal knowledge is culturally appropriate [23], but it can be complemented with Western medicine. Some authors have emphasized the positive view that traditional healers have towards Western medicine and their eagerness to cooperate with doctors [18-22]. Research in Bolivia [22], Nigeria [18], and Iran [20] suggests that cooperation between ethnomedicine and biomedicine is possible and might benefit local populations and their environment, whereas lack of cooperation can generate lack of understanding of biomedicine and lead to its misuse [24,25]. By analyzing how indigenous peoples conceptualize local and Western medicine, medical practitioners might be able to use biomedicine in a more culturally appropriate way.

### Objectives

In this research we study how the Tsimane', a native Amazonian population in Bolivia, conceptualize and use local and Western forms of medicinal knowledge. Specifically, we assess (1) whether the Tsimane' separate or integrate local and Western medicine, both conceptually and practically, and (2) whether practitioners of local and Western medicine (i.e., traditional healers and medical doctors) show readiness to cooperate to combine the two medical systems in their daily practices.

From the many domains of indigenous knowledge (i.e., medicine, ecology, astrology), we focus on local medicinal knowledge because a) 70–80% of the population in the developing world depends on medicinal plants for primary health care [19,23,26], and b) empirical studies suggest that local medicinal knowledge might be associated with better health and nutritional status [27-29].

We use the term local medicinal knowledge to refer to the cumulative body of knowledge of medicinal plants and healing practices of a culture that has been handed down through generations, that is socially shared by the members of the same generation, and that has been adapted to a particular place [10,30]. Under local medicinal knowledge we include the knowledge of the raw material from which remedies are produced and the socio-medical aspects implied in their preparation and uses [31]. We use the terms local practitioners to refer to traditional healers (people who use local medicinal knowledge) and health assistants (villagers trained to voluntarily oversee villager's health). Traditional healer is the best word we have found in English to refer to the Tsimane' term *cocojsi *or to the Spanish word *curandero*, the words used by Tsimane' to refer to native healers. We use the term Western medicine to refer to biomedicine and the term doctor to refer to people formally trained in Western medicine even if they do not have a medical degree (e.g., nurses). We use the term doctor to ease the narrative and because Tsimane' themselves use this word to refer to any partitioner of Western medicine. We use the word affections (rather than the word diseases) because when asked about the diseases suffered, the Tsimane' reply with symptoms not with the name of diseases, and we do not have the medical training to diagnose diseases.

The empirical analysis focuses on four gastrointestinal affections (stomachache, diarrhea, vomiting, and intestinal parasites) for three reasons. First, the incidence of gastrointestinal affections is high among the Tsimane' [24,25]. Second, gastrointestinal affections are recognized both by local and Western medicine and have well-identified symptoms. Selection of affections that are well recognized by the two medical systems allows us to overcome the inability of the Western medical system to identify social, cultural, and behavioral factors as causes of spiritual diseases. Third, gastrointestinal affections have a clear etiology among the Tsimane', who believe that some gastrointestinal ailments (diarrhea and intestinal parasites) can result from common causes (i.e. food poisoning) whereas others (stomachache and nausea) can result from common causes or from witchcraft.

## The Tsimane' and their medical system

The Tsimane's are the third largest ethnic group in the lowlands of Bolivia, with about 8,000 people [32]. The Tsimane' live in the Ballivian and Yacuma Provinces of the Department of Beni, and their territory spreads from the foothills of the Andes to the northeast, and reaches the edges of the Moxos savanna. The Tsimane' have traditionally been a semi-nomadic group [33] but currently reside in villages along river banks and logging roads. Villages in this study had an average of 24 nuclear households (Standard deviation (SD) = 10.88).

The Tsimane' are considered a foraging-horticultural society because of their heavy reliance on forest goods and agricultural crops in household consumption [34]. Nevertheless, Tsimane' are now taking up other occupations such as wage labor in logging camps, cattle ranches, and in the homesteads of colonist farmers [24,25,35]. Further reading on Tsimane' culture and society can be found in Ellis [33], Daillant [36] and Huanca [37]. Below we provide descriptions of topics related to the analysis presented in this paper, such as Tsimane' health, Tsimane' explanations of illnesses, availability of medical treatments and use of medicine among the Tsimane'.

### Tsimane' health

Previous research [25,39] suggests that infectious affections are an important health problem among the Tsimane', and that respiratory and gastrointestinal affections are the most commonly reported illnesses among both Tsimane' children and adults [25]. Intestinal parasites, especially hookworm, a soil-transmitted helminth, are endemic [24] and can cause stunted growth, a common condition among the Tsimane' [39].

Acculturation and access to market goods have the potential to deteriorate Tsimane' health in the short term for at least two reasons. First, previous research has shown that Tsimane' misuse Western medicine [24]. Most Tsimane' do not have the skills to select appropriate drugs without relying on advice from traders and doctors and they lack knowledge about pharmaceutical treatments. As a consequence, Tsimane' often do not use the appropriate drugs for their condition or use inappropriate dosages. Second, acculturation might generate loss of local medicinal knowledge, which seems to protect Tsimane' health and nutritional status [27-29]. Despite lack of empirical evidence on the topic [40], Tsimane' feel that they are loosing local medicinal knowledge, and that the young are not adequately learning this knowledge.

### Tsimane' explanations for the causes of illnesses

As other native Amazonian societies, Tsimane' consider that the world is divided between the natural tangible environment and the supernatural or spiritual realm [37,41]. This duality is embedded in the Tsimane's interpretation of the world [42] and can be found in Tsimane' explanations of the causes of illnesses.

The Tsimane' distinguish between common and spiritual illnesses [41]. Common illnesses (*japacjodye *in Tsimane' language) result from external or internal loci of cause, such as contact with hazardous agents or internally-initiated imbalances. Spiritual illnesses result from the purposeful intervention of a human or supernatural proxy. Spiritual illnesses are called *"*bush illnesses" (*däräcansi *in Tsimane') and are the result of witchcraft by malevolent spirits (or people) or the guardians of the natural environment (*jichi*) [41].

### Medical treatments available to the Tsimane'

In the event of an illness, Tsimane' use medicinal plants or Western medicines or consult a traditional healer. The Tsimane', who hold an extensive body of ethnobotanical knowledge, self-medicate with plant remedies. Previous research shows that from the many useful plants known to the Tsimane', medicine is the category in which Tsimane' recognize the largest number of useful plants (n = 169, 41%) [43]. Tsimane' believe in the curative power of plant remedies and the use of medicinal plants is popular, especially in isolated villages [43]. Tsimane' argue that the use of medicinal plants is decreasing due to the effort required to prepare plant remedies. Tsimane' said that gathering and preparing medicinal plants requires more effort and time than obtaining Western pharmaceuticals. Tsimane' also say that the curative power of plant remedies is slower than the curative power of pharmaceuticals. Lastly, Tsimane' say that some curative plants come with taboos and restrictions that the patient needs to follow for the plants to work well, and these cultural restrictions constitute added costs of medicinal plants.

Pharmaceuticals from pharmacies or stores in local market towns and from itinerant traders who visit Tsimane' villages are also available to the Tsimane'. Tsimane' can also ocasionally obtain free Western medicine from other visitors to their villages, i.e. researchers, vaccination campaigns. Although Tsimane' use of Western treatments has increased during the last 50 years in response to increasing exposure to Bolivian national society, pharmaceuticals are still less popular than plant remedies due to the long distances to pharmacies and the relative high price of pharmaceuticals for a population with low cash income. Missionaries introduced the first hospitals and drugs in the area during the second half of the 20^th ^century [56]. Nowadays, Tsimane' have access to a hospital in the town of San Borja and to a free clinic run by Missionaires in the vicinity of San Borja [44]. Tsimane' needing medical attention can use those facilities. Family members are expected to accompain the sick person and provide some work for the Missionaires to pay for the expenses.

Last, Tsimane' can consult traditional healers or other local experts who treat common and spiritual illnesses. Traditional healers can cure common diseases and are the only ones who can cure spiritual illnesses [41]. Byron [25] states that consulting traditional healers is more economical than going to the hospital, and this might be an important incentive for the Tsimane' to visit them.

### Tsimane' use of medicine

Tsimane' consider that the cause of an illness determines the appropriate treatment. Common illnesses, caused by the natural world, can be cured with medicinal plants or with Western medicines, whereas illnesses caused by spiritual beings can only be cured by traditional healers [41].

Sickness is first treated as a common, not as a spiritual, illness, and plant or pharmaceutical remedies are administered sequentially or simultaneously according to the symptoms of the patient. Tsimane' often self-medicate and they typically stop Western or local treatments once the disease symptoms disappear without necessarily following the full dose of treatment. If the condition persists, after self-medication, the Tsimane' typically ask for the advice of knowledgeable people in the village (typically elders). The Tsimane' begin to suspect that the illness might be caused by witchcraft if the person does not improve after several treatments. In this case, they seek the help of a traditional healer. Hospitals are only visited as a last resort, and mistrust on Western doctors is common among the Tsimane' [24,25].

## Methods

For background information, we relied on TAPS data [38]. Further information was gathered during June-July 2007 in three Tsimane' villages along the Maniqui River, province of Beni, Bolivia. PhD students in environmental sciences and cultural anthropology who were taking part in a summer training camp in methods of data collection helped in data collection. The research team lived in the villages described below in houses owned by the Tsimane' Amazonian Panel Stutdy. Three experienced Tsimane' translators helped to conduct the interviews in Tsimane' and served as key study participants.

### Site and sample

Participants for the study included people over the age of 16 in three Tsimane' villages: Yaranda, Santa María, and San Juan de Nápoles (hereafter Nápoles). We included people over the age of 16, because Tsimane' enter adulthood at that age. Young men and women of this age can marry, plant their own plot, and are supposed to be able to form a family. Two of the villages selected, Yaranda and Santa María, are far from the market town and only accessible by a motor canoe. Yaranda has 35 households and Santa Maria has 32 households. Nápoles lies about two hours by car from San Borja and has 14 households. Nápoles differs from Yaranda and Santa María because residents in Nápoles have easier access to the hospital in the outskirts of the local town of San Borja. Villagers from Nápoles also have less access to the forest due to increasing cattle ranching in the area and therefore less availability of medicinal plants.

We used different sampling strategies for each method of data collection described below. We used a snowball sample for the free listings. In San Borja we started free listing by interviewing a nurse who has been collaborating with the TAPS project for several years, and suggested the names of six study participants for free listings. In Yaranda, we started by asking our key study participants to identify the village traditional healer or other people with knowledge of medical treatments (n = 12). The total sample for the free listing exercise was 18. We used a purposive sample for semi-structured interviews and pile sorts. We conducted semi-structured interviews with six women and eignt men from 16 to 75 years of age and pile sorts with 21 women and 18 men from 16 to 75 years of age. Lastly, the sample for the survey included all adults (or people over the age of 16) in the villages of Yaranda and Nápoles (n = 87; 44 women and 43 men).

### Methods of data collection

Methods of data collection included methods 1) to gather background information (participant observation, semi-structured interviews, and TAPS 2006 survey), 2) to assess whether Tsimane' integrate local medicinal knowledge and Western medicine at the conceptual level (free listings and pile sorts), 3) to assess to what extent Tsimane' combine local medicinal knowledge with Western medicine in actual treatments (survey), and 4) to assess the willingness of Tsimane' and Western medical specialists to cooperate with each other (workshop).

#### Participant observation

We used participant observation [45] to achieve an understanding of the diseases, treatments, and relations between different bodies of knowledge. For example, upon request of the Great Tsimane' council and the villagers, we provided Western medicines to people who came to us when they were sick. A student with basic medical training was in charge of providing first aid. Interactions with sick people allowed us to observe Tsimane' requests for Western medicines. Those interactions also allowed us to gain a better understanding of Tsimane' diseases and treatment conceptualization.

#### Semi-structured interviews

We used semi-structured interviews [46] to gather information on local medicinal knowledge, the causes of diseases, the use of medicines, and the potential interest of local and Western medical professionals in cooperating with each other. Semi-structured interviews lasted less than one hour and were done with the help of a translator.

#### TAPS 2006 survey

We used a survey of the Tsimane' Amazonian Panel Study [38] to identify the main affections self-reported by people over the age of 16 (n = 679). The survey took place in 2006 during the dry season, which lasts from June to September. Previous work suggests no markedly seasonal changes in objective or self-reported ailments, and thus it is assumed that the answers obtained for the dry period reflect health during the entire year [24,25]. We found that 65% of people surveyed reported at least one ailment during the seven days before the day of the interview. The most common affection reported was the flu, followed by gastrointestinal affections. Fifteen percent of the participants reported having suffered from stomachache, diarrhea, vomiting, and intestinal parasites during the seven days before the day of the interview. So, we selected gastrointestinal affections as the focus of this study.

#### Free listing

We asked study participants to list all the medical treatments they knew to treat gastrointestinal affections [47,48]. We instructed participants to include in their lists both local and Western treatments. To get a better understanding of the full range of treatments available to Tsimane', we asked doctors and Tsimane'to list medical treatments. The final lists included local and Western treatments.

#### Pile sorts

From the list generated with the free listing technique, we selected the ten most frequent Western treatments and the ten most frequent plant treatments available within three hours walking from the village. The final list included nine plants, one mineral, and ten pharmaceutical drugs. To facilitate the identification of the items for the pile sort, we collected the plant species in the forest and bought the drugs selected from a pharmacy. We presented participants with the 20 items and asked them to sort the items in similarity groups [48,49]. After they had placed items in piles, we asked them to explain their reasons for placing items in a pile.

#### Survey

We constructed a survey using the insights of background information. The survey included socio-demographic and health questions. We asked all adults in the sample about the name of diseases (if any) suffered the week before the day of the interview and the name of the first three treatments used for any gastrointestinal affection suffered during the same period.

#### Workshop

At the end of the research, we organized a participatory workshop [50], which had three aims: (1) to explain the findings of our own research, (2) to identify the main threats to Tsimane' health, as perceived by Tsimane', and (3) to assess the willingness of Western and local medical practitioners to cooperate with each other. We invited three doctors, one nurse, four traditional healers, and four health assistants to the workshop. However, more health assistants arrived due to interest in the workshop, so the final number of participants included 34 persons. We invited these people for practical reasons. Two of the doctors and the nurse have previously worked with Tsimane'. The third doctor was very interested in the workshop. We also had previous contacts with the traditional healers and health assistants initially selected

### Analysis

We analyzed data from free listing and pile sorting using ANTHROPAC 4.983/X for Windows [51]. From responses to free listing, we calculated: 1) the percentage of people who mentioned each item, 2) the average rank of the order of mention of each item, and 3) the saliency of each reason (the weighted average of the inverse rank of an item across multiple free lists, where each list is weighted by the number of items on the list) [46]. The saliency index evaluates, with a range from 0 to 1, the overall importance of an item across all of the lists. The analysis of free listing allowed us to identify the most common treatments reported for the gastrointestinal affections selected.

We used non-metric multidimensional scaling (MDS) to analyze pile sort data. The MDS permits an observational assessment of whether people agree in the way they sort medical treatments. The closer the items are in the MDS, the more times they were classified together in individual pile sorts.

We used STATA 9 to analyze survey data. We calculated frequency of gastrointentinal affections and treatment options.

### Limitations

This study has two main limitations. First, data was collected with the help of translators. The use of translators for interviews contains the obvious challenge of language barriers and the possible loss of information. Second, since we limited our study to gastrointestinal affections, the selection of ailments could bias results. It is possible that results found in this research apply to gastrointestinal affections, but not to other diseases.

## Results

### Tsimane' conceptualization and use of local and Western medicine

Results from free lists and pile sorts suggest that Western treatments do not belong to the Tsimane' concept of medical treatments. Tsimane' participants in free listing listed 16 different treatments for gastrointestinal affections, none a Western treatment (Table 1). On average each informant listed 5.5 different treatments for gastrointestinal affections (SD = 2.4). The shortest list had two items and the longest nine. Oveto' (*Uncaria guianensis*) was singled out as the most important and salient item in the lists. The 12 study participants interviewed for free listing mentioned Oveto', and most mentioned Oveto' as the first item in their lists. Eleven of the 16 treatments that appeared in free listings were listed by two or more people and five treatments were listed only by one person.

**Table 1:** Ten most frequent items reported in free listings of gastrointestinal treatments by Tsimane' study participants (n = 12).

**Scientific name^a^**	**Family**	**Tsimane'** ** name**	**Voucher**	**Frequency**	**Salience^b^**	**Gastrointestinal** ** affection treated**
Uncaria guianensis *(Aubl.) Gremlin*	Rubiaceae	Oveto'	TH044	12	0.29	Stomachache and diarrhea
*Galipea longiflora*	Rutaceae	Ibam'ta	TH257	9	0.17	Stomachache and vomiting
*Aspidosperma rigidum*	Flacourtiaceae	Vambason	TH153	5	0.13	Stomachache and diarrhea
Alum. Double sulfate of Aluminum and Potassium		Curpa		5	0.09	Stomachache and vomiting
*Ficus cf. insípida *Willd	Moraceae	Titij'	TH123	5	0.15	Intestinal parasites
*Hymenaea courbaril *L.	Leguminosae-Pap	Vejqui'	TH072	5	0.12	Stomachache and diarrhea
*Carica papaya*	Caricaceae	Pofi		3	0.10	Intestinal parasites
Indetermined		Jamo'tarara		3	0.08	Stomachache and intestinal parasites
*Citrus lemon*	Rutaceae	Ashasha	TH531	3	0.10	Diarrhea and vomiting
*Sparattanthelium burchellii *Rusby	Hennandaceae	Vayori	AN023	3	0.04	Diarrhea
Responses per person	Mean = 5.5					
	Minimum = 2					
	Maximum = 9					
	SD = 2.4					

Notes: ^a ^For this research, we did not collect vouchers. Information on scientific name and vouchers commes from TAPS data. Vouchers specimens are deposited in the Herbario Nacional de Bolivia, Universidad Mayor de San Andrés, La Paz.

^b ^Salience (S) takes into account the frequency (F) of a given item in lists and the average rank of the item in the respondents list.

S = F/NmP

**Figure 1:**
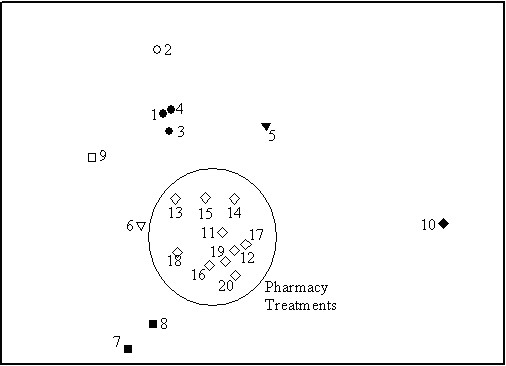
**Non-metric multidimensional scaling with data from pile sorts of ethnomedical and biomedical treatments for gastrointestinal affections**. Vejqui'(1), Vuayuri (3), Oveto'(4). Ibam'ta (2). Vambason (5). Mana'i root (6). Titij'(7), Pofi seed (8). Ashasha (9). Curpa (10). Klosidol (11), Viadil (12), Metronidazol (13), Carbon extract (14), Amoxiciline (15), Domper (16), Metoclopramida (17), Mebendazol (18), Albendazol (19), Noxon (20).

Results from pile sorts concur with results from free listings and further confirm Tsimane' conceptual divide between local and Western medicine. Figure 1 shows the results from a non-metric multidimensional scaling with data from pile sorts from participants in three communities. Tsimane' placed the 20 medicinal items into three categories:1) medicinal plants to cure gastrointestinal affections, 2) a mineral, curpa, used to cure illnesses caused by sorcery, and 3) pharmaceutical treatments that doctors recommend to cure gastrointestinal affections.

Among the nine medicinal plants used in the pile sorting exercise, we identify six distinct groups. Information from interviews after pile sorting revealed that Tsimane' classify medicinal plants according to the affection that the plant can cure. For example, the Tsimane' pile together *vejqui *(1)*', vuayuri *(3), and *oveto' *(4) because they use them to cure diarrhea and stomachache. *Ibam'ta *(2) a plant that is used to treat stomachache and leishmaniasis (not a gastrointestinal disease) appears close to the group of plants to cure stomachache. Similarly, Tsimane' use *vambason *(5), *mana'i *root (6), and *ashasha *(9) to treat gastrointestinal and other diseases. For example, *vambason *is used with kidney problems, *ashasha *is used for colds, and *mana'i *is used to cure intestinal parasites. Since Tsimane' could only put an item into only one pile, these items appear separate from one another. Tsimane' use *titij' *(7) and *pofi *seeds (8) to treat intestinal parasites; for this reason they lie next to each other in Figure 1.

In Figure 1 the mineral, *curpa*, stands apart from both plants and Western medicines. *Curpa *is used to heal spiritual illnesses regardless of the symptoms. Lastly, we found a single cluster that grouped all the items belonging to the list generated by Western medical practitioners. Tsimane' put all pharmaceutical treatments in a single group. During interviews after the pile sorting exercise, when we asked participants the reasons for their classification, they answered that they put pharmaceutical treatments together because they did not know their exact use.

To assess whether the results from MDS were similar in the three studied villages, we ran three non-metric multidimensional scaling with data from each village separately (results not shown). We found no substantive differences across villages regarding the classification of plant remedies, but we found some differences regarding the classification of Western remedies. In the most isolated village, Yaranda, people put Western treatments in a unique category (unknown), whereas in the other two communities, Santa Maria and Nápoles, people recognized at least four uses for Western remedies: headache, toothache, flu, and cold.

Results from the survey suggest that, at the practical level, Tsimane' mix local and Western treatments to cure gastrointestinal affections. Of the 87 people interviewed, 64 (73.5%) reported having been sick the week before the interview. We analyzed information from the first ailment reported and found that the most common illnesses reported were cold (20.7% of the people who reported any ailment), headache (9.2%), and diarrhea (9.2%).17.2% of the people who reported any ailment suffered from gastrointestinal affections, including diarrhea (9.2%), stomachache (6.9%), and vomiting (1.1%). None of the respondents reported suffering from intestinal parasites. The distribution of ailments resembles the distribution of ailments reported by Byron [25] for the same population.

**Figure 2:**
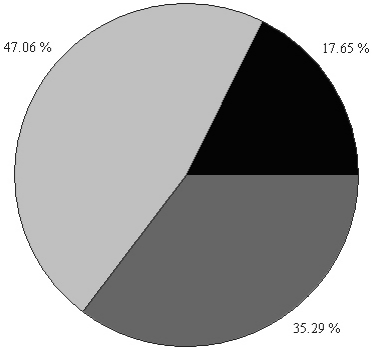
**Reported frequencies of treatments options for gastrointestinal affections during the week before the interview (n = 18)**. Pharmaceutical treatment (17.65%). Pharmaceutical and plant treatments (47.06%). Plant treatment (35.29%).

From the people surveyed (n = 87), 18.2% did not use any medicine to cure themselves. From the ones who used some treatment (Figure 2), 35.3% used plant treatments only and 17.6% used pharmaceutical treatments only. The remaining 47.1% of the people who used any treatment to cure gastrointestinal affections combined pharmaceutical and plant treatments.

The results also suggest that Tsimane' first rely on local medicinal knowledge to treat gastrointestinal affections. From the people who took any treatment for gastrointestinal affections (n = 52), 76.5% chose medicinal plants first and only 23.5% chose a pharmaceutical treatment first.

### Assessing the willingness to cooperate

In semi-structured interviews, doctors and local practitioners emphasized the need for a medical system that allowed for cooperation between local and Western medicine. Doctors and local practitioners agreed that some diseases (i.e., tuberculosis) should be treated with Western medicines, whereas other affections (e.g. mild diarrhea) could be better treated with local plant-based medicines. There was also broad agreement that a system which allowed for the collaboration of local and Western medicine would give patients the best treatment for their disease. They also expressed a desire to learn one from each other, although doctors mistrusted the medicinal value of plants until they could be scientifically validated.

To probe the willingness to cooperate, we organized a workshop with doctors and local practitioners. Participants discussed the advantages of both medical systems, arguing that while the local medical system was cheap and popular, Western medicine was effective and endorsed by scientific studies.

Participants in the workshop felt that health was a high priority for them and presented ideas to improve it. Some of the ideas included: (1) to train health assistants in local and Western medicine, (2) to strengthen local medicinal knowledge, (3) to strengthen the role of traditional healers and health assistants, (4) to open medical posts in communities, (5) to improve education and prevention of diseases, (6) to take care of the natural environment, (7) to build communal gardens of medicinal plants, and (8) to build physical infrastructure that might have a preventive impact on health, such as wells or latrines.

Participants discussed the case of Apillapampa (Cochabamba, Bolivia) where a traditional healer works in conjunction with the system of Western health care [22]. The reappraisal of local medicinal knowledge turned into one of the main points of discussion of the workshop since participants had the impression that, in the more isolated communities, plant remedies were the main health resource available. Participants also felt that they could obtain an economic benefit with the revalorization of medicinal plants and also contribute to the conservation of forest lands. Participants considered that organization of training workshops, valorization of elders, cooperation with doctors, procurement of institutional help, and the maintenance of customs could help achieve the revalorization of local medicinal knowledge.

Participants viewed the workshop as the first spark to encourage the interaction between doctors and local practitioners and highlighted their willingness to continue cooperating. They proposed immediate steps such as the construction of school gardens with medicinal plants; the sharing of elders' medicinal knowledge in schools, and the compilation of local medical treatments within their communities. Participants also proposed to ask the municipal government for money to conduct more workshops in which the relations between local and Western medicine could be further discussed.

## Discussion

Three main substantive findings emerge from this work. First, Tsimane' do not include Western medicine in their conceptualization of medical treatments. Second, at the practical level, Tsimane' do mix local and Western treatments, although the frequency of use of local treatments is higher than the frequency of use of Western treatments. Last, doctors and local health practitioners expressed willingness to cooperate to improve the health of Tsimane'.

The first substantive finding, that Tsimane' do not include Western remedies in their conceptualization of medical treatments, fits with findings from another case study among the Tsimane' [52]. In an ethnobotanical study in the village of Tacuaral de Matos (35 kilometers away from San Borja), Ticona found that Tsimane' do not include pharmaceutical treatments in the category of *pinidyedyes *(medicine), although they interchangeably use the Tsimane' word *pinidye*' and the Spanish words *remedios *(treatments) or *medicinas *(medicine) to acquire drugs from traders or in pharmacies. A possible explanation of this finding is that since illness is a universal and recurrent problem and health treatments within a village are usually limited, villagers might develop shared standards of treatment [53] such as particular plants to cure gastrointestinal ailments. Tsimane' still have limited access to Western treatments due to distance from pharmacies and hospitals and low cash income. These limitations together with the short history of Western treatments in the area might explain why Tsimane' do not include Western treatments in their concept of medical treatments.

We also found that Tsimane' do not have distinct categories for Western treatments. The finding has important implications for the use of Western medicine, and dovetails with ethnographic information about Tsimane' misuse of Western treatments. Because Tsimane' do not have a classificatory system for Western medical treatments, they use them independently of the ailment suffered. Tsimane' typically do not rely on an individual trained in Western medicine to provide instructions on how to take Western medicines. Because of the many dangers associated with the misuse of pharmaceutical treatments, the lack of categorization of Western medicines might have pernicious effects on Tsimane' health [24].

The second substantive finding is that in practice Tsimane' do combine local and Western medicines. Medical pluralism occurs in many settings and provides insights into connections between health, knowledge, treatment behavior, and the cultural significance of medicine [54,55]. For the Tsimane', the explanation fits with the complex systems of responses related to resources availability, cultural beliefs about illness origin, and personal interpretation of symptoms. For example, when asked for treatment preferences one informant said: "If I have money I buy tablets, but if I don't, I use medicinal plants. If nothing works, then I am probably sick with a bush illness. I rely more on medicinal plants than on drugs, but the bush is too far and the treatment too long" (personal interview 6/29/07).

Our results also suggest that Tsimane' still use medicinal plants more often than Western remedies as the first treatment for gastrointestinal affections. The prevalence of medicinal plants over Western medicines among the Tsimane' is consonant with findings among other indigenous groups. For example, in the village of Zapotitlán (Puebla, Mexico), Hernández et al. [57] found that 74% people interviewed used medicinal plant to treat gastrointestinal affections. In a study among the Caboclos in the Amazon estuary (Brasil), Reeve [55] also found that the principal method of self-treatment was medicinal plants. Both practical (i.e., accesibility) and cultural (i.e., taboos) reasons can account for this preference among the 'Tsimane.

The third finding from this research is that doctors and local practitioners felt that cooperation between them was important for Tsimane' health because the two medical systems could complement each other. Reeve [55] argues that amplifying the capacity of traditional healers to refer patients in need of clinical care, and clinician training for cooperation with traditional healers would assure more effective health care. Collaboration of the two medical systems would allow Tsimane' to choose the best treatment option for each ailment. Furthermore, doctors and traditional healers expressed a desire to learn more from each other. Understanding the points of articulation that develop between local healing and Western medicine within medically plural systems will contribute to the development of more integrative models of cooperation [55].

## Conclusion

In this article we have analyzed Tsimane' treatment system for gastrointestinal affections. We have found that the Tsimane' conceptualize local and Western medicine as two independent domains of knowledge, although they mix pharmaceutical and plant treatments in their daily practice. We have also found that local practitioners and doctors show willingness to cooperate so people could benefit from both medical systems simultaneously.

Western medicine is regarded as having qualities of power, trust, and effectiveness by Tsimane' villagers and doctors. However a sense of revalorization of local medicinal knowledge is also present. Tsimane' feel that local medicinal knowledge helps to maintain the Tsimane' lifestyle and conserve the ecosystem. Current lack of access and poor understanding of the biomedical system may contribute to poor Tsimane' health. For this reason, the exploration of the connections between local medicinal knowledge and Western medicine and the possible co-management of health among traditional healers and doctors could lead to an improvement in the health situation as well as the conservation of their own ecosystem. As Chapman [58] argues, local medicinal knowledge should not be fully integrated into science, nor should the reverse occur. Both are complementary, not replaceable. Both have value in their own right and need to be recognized as such, giving both equal weights.

At this point what remains to be answered is how this cooperation could be effective. There is a need to further analyze the policy for implementing cooperation. This is particular relevant in a context where local medical system and Western medical system are not viewed as being of equal worth, with Western practitioners frequently expressing disdain for non-Western treatment options. Some ideas have emerged from this research, however further study is required in order to achieve the equitable cooperation of both medical systems.

## Competing interests

The authors declare that they have no competing interests.

## Authors' contributions

LCM collected and analyzed data and wrote the article. VRG and ST contributed to analysis and interpretations of data and critically reviewed the manuscript. All authors read and approved the final manuscript.
